# Jasmonate regulates plant resistance to *Pectobacterium brasiliense* by inducing indole glucosinolate biosynthesis

**DOI:** 10.3389/fpls.2022.964092

**Published:** 2022-09-29

**Authors:** So Young Yi, Myungjin Lee, Sun Kyu Park, Lu Lu, Gisuk Lee, Sang-Gyu Kim, Si-Yong Kang, Yong Pyo Lim

**Affiliations:** ^1^ Institute of Agricultural Science, Chungnam National University, Daejeon, South Korea; ^2^ Molecular Genetics and Genomics Laboratory, Department of Horticulture, Chungnam National University, Daejeon, South Korea; ^3^ Department of Biological Sciences, Korea Advanced Institute for Science and Technology, Daejeon, South Korea; ^4^ Department of Horticulture, College of Industrial Sciences, Kongju National University, Yesan, South Korea; ^5^ Research Center of Crop Breeding for Omics and Artificial Intelligence, Kongju National University, Yesan, South Korea

**Keywords:** *Brassica rapa*, flg22-triggered immunity, jasmonic acid, necrotrophic bacteria, bacterial soft rot, indole glucosinolate, neoglucobrassicin, *Pectobacterium brasiliense*

## Abstract

*Pectobacterium brasiliense* (*P. brasiliense*) is a necrotrophic bacterium that causes the soft rot disease in *Brassica rapa*. However, the mechanisms underlying plant immune responses against necrotrophic bacterial pathogens with a broad host range are still not well understood. Using a flg22-triggered seedling growth inhibition (SGI) assay with 455 *Brassica rapa* inbred lines, we selected six *B. rapa* flagellin-insensitive lines (*Br*fin2-7) and three *B. rapa* flagellin-sensitive lines (*Br*fs1-3). *Br*fin lines showed compromised flg22-induced immune responses (oxidative burst, mitogen-activated protein kinase (MAPK) activation, and seedling growth inhibition) compared to the control line R-o-18; nevertheless, they were resistant to *P. brasiliense*. To explain this, we analyzed the phytohormone content and found that most *Br*fin lines had higher *P. brasiliense*-induced jasmonic acid (JA) than *Br*fs lines. Moreover, MeJA pretreatment enhanced the resistance of *B. rapa* to *P. brasiliense*. To explain the correlation between the resistance of *Br*fin lines to *P. brasiliense* and activated JA signaling, we analyzed pathogen-induced glucosinolate (GS) content in *B. rapa*. Notably, in *Br*fin7, the neoglucobrassicin (NGBS) content among indole glucosinolates (IGS) was significantly higher than that in *Br*fs2 following *P. brasiliense* inoculation, and genes involved in IGSs biosynthesis were also highly expressed. Furthermore, almost all *Br*fin lines with high JA levels and resistance to *P. brasiliense* had higher *P. brasiliense*-induced NGBS levels than *Br*fs lines. Thus, our results show that activated JA-mediated signaling attenuates flg22-triggered immunity but enhances resistance to *P. brasiliense* by inducing indole glucosinolate biosynthesis in *Brassica rapa*. This study provides novel insights into the role of JA-mediated defense against necrotrophic bacterial pathogens within a broad host range.

## Introduction

Plants have developed complex defense systems against invading pathogens. The first line of plant defense is activated by the perception of pathogen-associated molecular patterns (PAMPs) such as flagellin, lipopolysaccharide, peptidoglycan, and EF-Tu, which are recognized by plant pattern recognition receptors (PRRs) (Abramovitch et al., 2006; Jones and Dangl, 2006; Zipfel, 2014). PAMP-triggered immunity (PTI) leads to a series of host responses, including oxidative burst (Jones and Dangl, 2006; He et al., 2007), stimulation of mitogen-activated protein kinase (MAPK) cascades (Asai et al., 2002), transcriptional reprogramming, and cell wall reinforcement *via* callose deposition (Nishimura et al., 2003). Plants perceive effectors through resistance (R) proteins and activate a robust and rapid defense response, namely effector-triggered immunity (ETI) (Alfano and Collmer, 1997), which leads to programmed cell death in the local tissue (Dangl et al., 1996). Moreover, plants undergo transcriptional reprogramming to activate the expression of defense genes (Caplan et al., 2008; Bhattacharjee et al., 2013).

*P. brasiliense*, which is a necrotrophic bacterial pathogen from the family Pectobacteriaceae (Onkendi et al., 2014), is responsible for several serious pre- and post-harvest diseases of various plant types worldwide (Toth et al., 2003). Resistance to *P. brasiliense*, which has a broad host range, may be explained by PTI rather than by the expression of single resistance genes. The phytopathogenicity of *P. brasiliense* is largely related to its ability to synthesize and secrete plant cell wall-degrading enzymes (PCWDEs), including pectinases, cellulases, and proteases (Pirhonen et al., 1993; Heikinheimo et al., 1995; Mae et al., 1995; Marits et al., 1999). Typical symptoms of *P. brasiliense* infections include maceration and rotting of the leaves and other plant organs (Davidsson et al., 2013). Plant cell wall fragments released by PCWDEs secreted by *P. brasiliense* can act as danger-associated molecular patterns (DAMPs) that are recognized by PRRs to activate PTI in response to an invading pathogen (Mengiste, 2012). However, ETI has not been reported for necrotrophic bacterial pathogens, and host cell death is not expected to restrict necrotrophic pathogen growth (Glazebrook, 2005).

Phytohormones are crucial regulators of plant immune responses. Both ETI and PTI involve signaling pathways associated with common phytohormones, including salicylic acid (SA), jasmonic acid (JA), and ethylene (ET) (Glazebrook, 2005; Tsuda and Katagiri, 2010; Pieterse et al., 2012; Yi et al., 2014). The JA pathway generally protects against necrotrophic pathogens, whereas the SA pathway is associated with plant resistance to biotrophic pathogens (Glazebrook, 2005; Bari and Jones, 2009; Ali et al., 2017). Regarding *Pectobacterium* species, JA/ET- and SA-mediated signaling positively affect defense responses (Palva et al., 1994; Vidal et al., 1997; Norman-Setterblad et al., 2000; Li et al., 2004; Liu et al., 2019; Tsers et al., 2020). Abscisic acid (ABA), an abiotic stress signal, is important for modulating diverse plant-pathogen interactions. Several studies have shown that ABA biosynthesis is required for effective disease resistance against necrotrophic fungal pathogens (Ton and Mauch-Mani, 2004; Adie et al., 2007; Garcia-Andrade et al., 2011).

Chemical β-aminobutyric acid (BABA) enhances *Arabidopsis thaliana* resistance to hemibiotrophic bacteria by priming the salicylic acid (SA) defense response (Zimmerli et al., 2000; Ton et al., 2005). BABA also primes the PTI response upon necrotrophic bacterial *P. brasiliense* infection BABA primed the expression of the PTI-responsive genes FLG22-INDUCED RECEPTOR-LIKE KINASE 1 (FRK1), ARABIDOPSIS NON-RACE SPECIFIC DISEASE RESISTANCE GENE (NDR1)/HAIRPIN-INDUCED GENE (HIN1)-LIKE 10 (NHL10), and CYTOCHROME P450, FAMILY 81 (CYP81F2); callose deposition; and boosted Arabidopsis stomatal immunity to *P. brasiliense*. (Po-Wen et al., 2013). Vitamin B6 is a potent antioxidant that helps plants cope with both biotic and abiotic stress conditions (Vanderschuren et al., 2013). VitB6 and its *de novo* and salvage biosynthetic pathways positively regulate defense responses against *P. brasiliense* by modulating cellular antioxidant capacity (Chandrasekaran and Chun, 2018). Plant ferredoxin-like protein is a ferredoxin-I protein that is involved in the hypersensitive response (HR) and plant immune response to bacterial pathogens (You et al., 2003). A recent report showed that PFLP intensifies disease resistance against bacterial soft rot through the MAPK pathway in PAMP-triggered immunity (Hong et al., 2018).

Glucosinolates (GS), which are unique secondary metabolites found in Brassicaceae species, have long been considered to contribute to plant-microbe interactions (Brader et al., 2001; Halkier and Gershenzon, 2006). Various molecular and genetic studies on Arabidopsis have identified most structural genes and transcription factors involved in GS biosynthesis (Hirai et al., 2007; Pfalz et al., 2009; Sonderby et al., 2010). Several studies have confirmed that tryptophan pathway genes involved in indole glucosinolate (IGS) biosynthesis are upregulated in *F. oxysporum*-infected plants (Kidd et al., 2011; Zhu et al., 2013), and the virulence of *Colletotrichum gloeosporioides* and *Colletotrichum orbiculare* is restricted by IGL in *B. rapa* (Hiruma et al., 2010). Bacterial pathogen inoculation also triggers upregulation of GS biosynthesis (Liu et al., 2019; Tinte et al., 2020). Furthermore, *CYP79* overexpressed in Arabidopsis showed increased GS synthesis and enhanced resistance to *P. brasiliense*, suggesting that altering GS profiles can modulate disease resistance in plants (Brader et al., 2006). Recent studies have shown that MYB34, MYB51, and MYB122 are involved in regulating IGS biosynthesis (Gigolashvili et al., 2007; Hirai et al., 2007; Gigolashvili et al., 2008; Sonderby et al., 2010) and mediate the biosynthesis of plant hormones involved in plant defense (Frerigmann and Gigolashvili, 2014). Additionally, Wiesner et al. (Wiesner et al., 2013) reported enhanced production of IGS by JA or MeJA treatment in *Brassica rapa*.

*P. brasiliense* is a necrotrophic bacterium that causes the soft rot disease in *Brassica rapa*. The effect of plant flg22 perception on *P. brasiliense* interactions remains unclear. Pretreatment of Arabidopsis with flagellin enhances the disease resistance of plants against hemibiotrophic bacterial pathogens (*Pst* DC3000) (Zipfel et al., 2004). However, it remains unclear whether flg22-triggered immunity is correlated with the extent of resistance to necrotrophic bacterial pathogens. The most *Br*fin lines selected through the SGI assay (the lines most insensitive to flg22) were relatively resistant to *P. brasiliense.* Furthermore, we revealed that pathogen-induced changes in the contents of JA and IGS were critical determinants of plant immunity in *B. rapa* infected with *P. brasiliense*. Our study demonstrated that flg22-triggered immunity differs between necrotrophic (e.g., *P. brasiliense*) and hemibiotrophic bacterial pathogens (*Pst* DC3000). Based on these results, the relative resistance of *Br*fin lines and accumulation of high JA against *P. brasiliense* may be explained by activated JA signaling, which suppresses flg22-triggered responses, whereas the tolerance of *B. rapa* to *P. brasiliense* is dependent on JA signaling.

## Results

### Growth inhibition by flg22 in a collection of diverse *B. rapa* inbred lines

**Figure 1:**
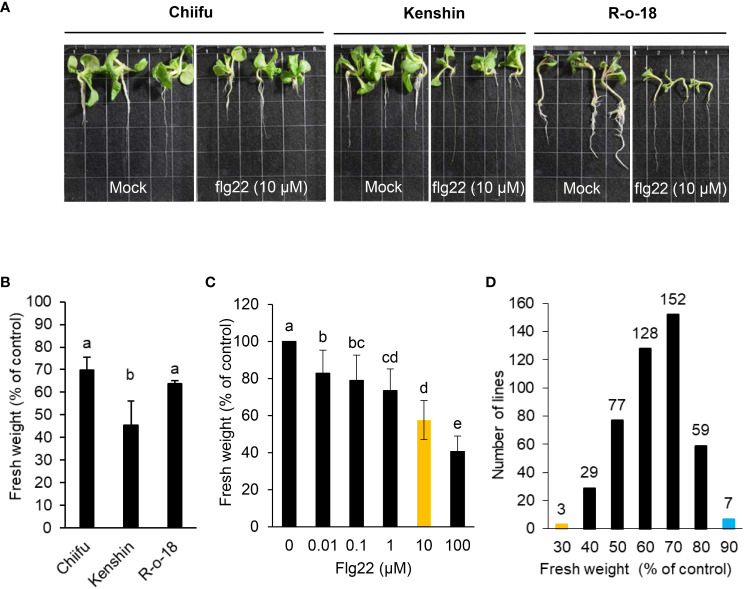
Effects of flg22 on the growth of *B. rapa* inbred lines. **(A, B)** Six-day-old seedlings of inbred lines Chiifu, Kenshin, and R-o-18 were incubated for an additional 6 days in plates containing Murashige and Skoog (MS) agar medium with or without 10 µM flg22. **(C)** Dose dependency of seedling growth inhibition. Six-day-old R-o-18 seedlings were incubated for an additional 6 days in liquid MS medium with or without flg22. Error bars represent standard deviations (n = 9 seedlings). **(D)** Six-day-old seedlings of 455 *B. rapa* inbred lines were incubated for an additional 6 days in liquid MS medium containing H_2_O or flg22 (10 µM). Different letters indicate significant differences among plant genotypes **(B)** or treatment **(C)**, respectively (α = 0.05, one-way ANOVA and Tukey’s HSD test; SPSS software).

**Figure 2:**
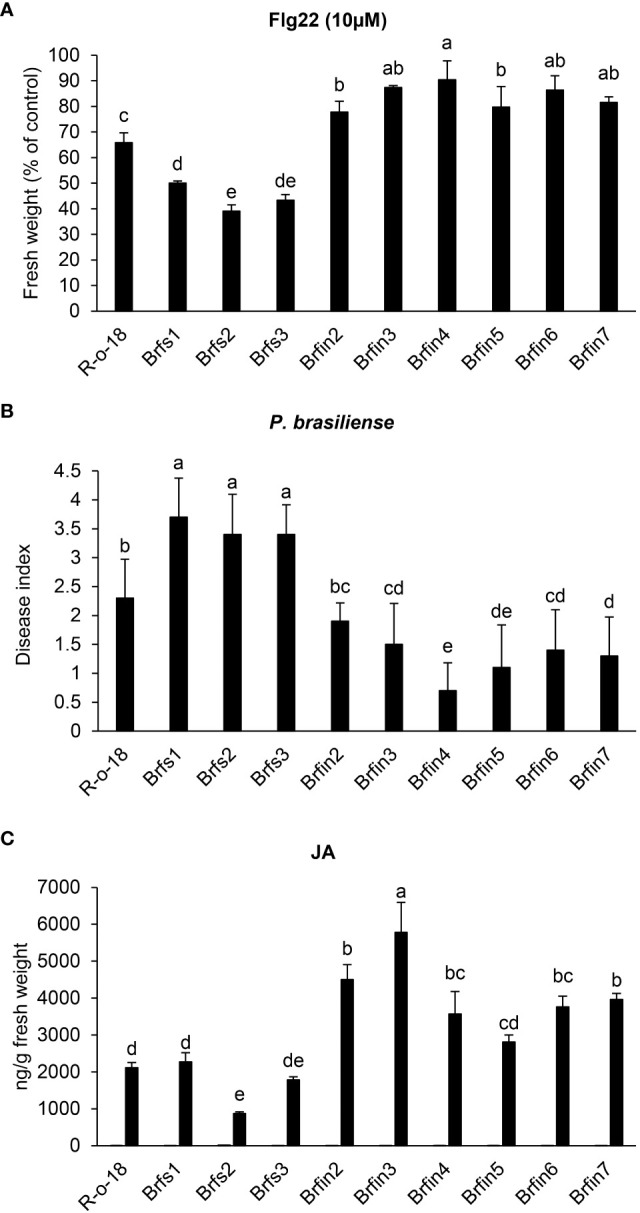
Phenotypes of *Br*fs and *Br*fin lines during flg22- or *P. brasiliense*- triggered signaling. **(A)** Effects of flg22 on the growth of *Br*fs and *Br*fin lines. Six-day-old seedlings *Br*fs, *Br*fin lines and R-o-18 were incubated for an additional 6 days in plates containing Murashige and Skoog (MS) agar medium with or without 10 µM flg22. **(B)** Relatively strong resistance of *Br*fin lines to the soft rot pathogen *P. brasiliense.* 24-day-old *B. rapa* plants were inoculated with *P. brasiliense* KACC 10225 by drenching the proximal plant parts with a bacterial suspension (1 × 10^6^ CFU/mL). At 7 days post-inoculation, disease severity was evaluated on a 0-4 scale and then converted to a percentage. Error bars represent standard deviations of 30 replications. Similar results were obtained in at least two independent experiments. Different letters indicate significant differences among plant genotypes (α = 0.01, one-way ANOVA and Duncan test; SPSS software). **(C)** Endogenous and *P. brasiliense*-induced phytohormone contents in *B. rapa* inbred lines. The JA contents in the control and infected leaves were quantified at 24 h post-infection. Error bars represent standard deviations of five replications. Similar results were obtained in at least two independent experiments. Different letters indicate significant differences among plant genotypes (α = 0.05, one-way ANOVA and Duncan test; SPSS software).

Host plants deploy different defense mechanisms and appropriate immune responses to defend themselves against necrotrophic pathogenic bacteria with a broad host range. Resistance to these pathogens is complex and does not appear to involve one resistance gene alone. *P. brasiliense* is a flagellated gram-negative bacterium. A potent PAMP, flg22, triggers immune responses in multiple plant species after it is detected by PRR FLAGELLIN-SENSING2 (FLS2). However, it is unknown whether flg22 detection by FLS2 in *B. rapa* leads to enhanced basal immunity (PAMP-triggered immunity, PTI) during *P. brasiliense* infection. To analyze the effect of flg22 on the interaction between *P. brasiliense* and *B. rapa*, we conducted a seedling growth inhibition (SGI) assay, one of the most sensitive and convenient assays for activating FLS2 following treatment with flg22. Among the 455 inbred *B. rapa* inbred in our collection, Chiifu (http://brassicadb.cn), R-o-18 (Stephenson et al., 2010) and Kenshin (Vanjildorj et al., 2009) have published genome information or functional studies (**Figures 1A–C**). We selected these three lines and used them to establish a system of SGI testing for *B. rapa*. We examined 455 *B. rapa* inbred lines to determine variations in flg22-induced growth inhibition. In most cases, the addition of flg22 adversely affected seedling growth (**Figure 1**). The flg22 treatment affected the root, leaf, and cotyledon growth of various *B. rapa* inbred (**Figure 1A**), resulting in a substantial decrease in fresh weight (**Figure 1B**). The inhibitory effect depended on the flg22 dose, with ~ 10 µM of flg22 leading to a half-maximal decrease in growth (**Figure 1C**). This high-throughput assay may be useful for the large-scale quantitative analysis of PTI in *B*. *rapa*. Of the 455 Chinese cabbage inbred lines, 280 lines, representing more than 60%, had flg22-induced SGI rates of 30–40%, as opposed to the untreated control plants (**Figure 1D**). *B. rapa* genotype R-o-18 is rapidly cycling and self-compatible. The flg22-triggered SGI rate of R-o-18 was also ~ 30%–40% (**Figure 1B**); hence, this line was set as the control line for the SGI assay system and used for comparison when selecting a line with an altered flg22-triggered SGI rate. Notably, seven *B. rapa* accessions were highly insensitive to flg22, with an SGI rate of less than 10% (**Figure 1D**). The seven lines were named *B. rapa* flagellin-insensitive (*Br*fin1-*Br*fin7). In contrast, three *B. rapa* accessions were highly sensitive to flg22, with an SGI rate of > 80% (**Figure 1D**). These lines were named *B. rapa* flagellin-sensitive (*Br*fs1-*Br*fs3). **Figure 2A** shows the repetitive significantly altered flg22-triggered growth inhibition rates of nine *B. rapa* inbred lines (three *Br*fs and six *Br*fin lines) among the lines selected in the first SGI assay. However, the Flg22 insensitivity in *Br*fin1was not reproduced in the second test, and the SGI rate was similar to that of the control line R-o-18.

### Flg22-insensitive lines exhibited increased resistance to *P. brasiliense*

To investigate whether flg22 sensitivity is correlated with plant resistance to *P. brasiliense*, the responses of *Br*fs and *Br*fin lines to inoculation with *P. brasiliense* (KACC 10225) were evaluated (**Figure 2B**). To assess the severity of the disease in *Br*fs and *Br*fin seedlings infected with *P. brasiliense*, macerated leaf lesions were scored according to a modified version of a previously described method (Lee et al., 2020) (see Materials and Methods). The disease index of R-o-18 inoculated with *P. brasiliense* was 2.3 (**Figures 2A, B**). Unexpectedly, six *Br*fin lines, which were most insensitive to flg22, had a disease index of 0.7–1.9. Among them, five *Br*fin lines tested in this experiment were significantly more resistant to *P. brasiliense* than to R-o-18 (**Figures 2A, B**). Additionally, the disease index of the three *Br*fs lines for *P. brasiliense* inoculation was > 3.5. Flg22 sensitive lines were significantly more susceptible to *P. brasiliense* than *Br*fin lines (**Figures 2A, B**). In the case of the interaction between *Pst* DC3000 and Arabidopsis, it has been reported that flg22 perception induces PTI, and the level of flg22-response sensitivity is associated with the enhancement of basal resistance (Trujillo et al., 2008). However, our results revealed that during *P. brasiliense* and Chinese cabbage interaction, sensitivity to flg22 was not related to the improvement of the basal resistance of plants.

### Comparison of phytohormone contents among selected lines (*Br*fs, *Br*fin lines, and R-o-18)

To help identify whether activated plant hormone signaling pathways contribute to the enhanced resistance of the *Br*fin lines to *P. brasiliense* (**Figure 2C** and **Supplementary Figure S1**), we analyzed the levels of plant hormones in *Br*fs, *Br*fin lines, and R-o-18. The ABA, SA, and JA contents were measured in *B. rapa* plants before and 24 h after *P. brasiliense* inoculation *via* LC-MS/MS analysis (**Figure 2C** and **Supplementary Figure S1**). The *P. brasiliense*-induced JA levels in the *Br*fin lines were ~ 140%–250% of those in R-o-18 (**Figure 2C**). ABA levels in *Br*fin lines (except *Br*fin3) were also higher than those in *Br*fs lines following *P. brasiliense* inoculation (**Supplementary Figure S1**). The relatively high pathogen-induced JA and ABA levels in most *Br*fin lines may explain the enhanced resistance of these lines to *P. brasiliense*. However, the *P. brasiliense*-induced SA levels in both the *Br*fs and *Br*fin lines were less than 50% of that of R-o-18, except for *Br*fin2 (**Supplementary Figure S1**). Additionally, we analyzed the basal levels of endogenous phytohormones before *P. brasiliense* inoculation. The basal levels of SA and JA were similar among *Br*fs, *Br*fin lines, and R-o-18, whereas the ABA level was more than 6-times higher in R-o-18 than in both *Br*fs and *Br*fin lines, except *Br*fs2 (**Figure 2C** and **Supplementary Figure S1**). These results suggest that endogenous basal levels of phytohormones (SA, JA, and ABA) may not affect plant basal resistance to *P. brasiliense*.

### Suppressed PTI responses in *Br*fin6 and *Br*fin7

**Figure 3:**
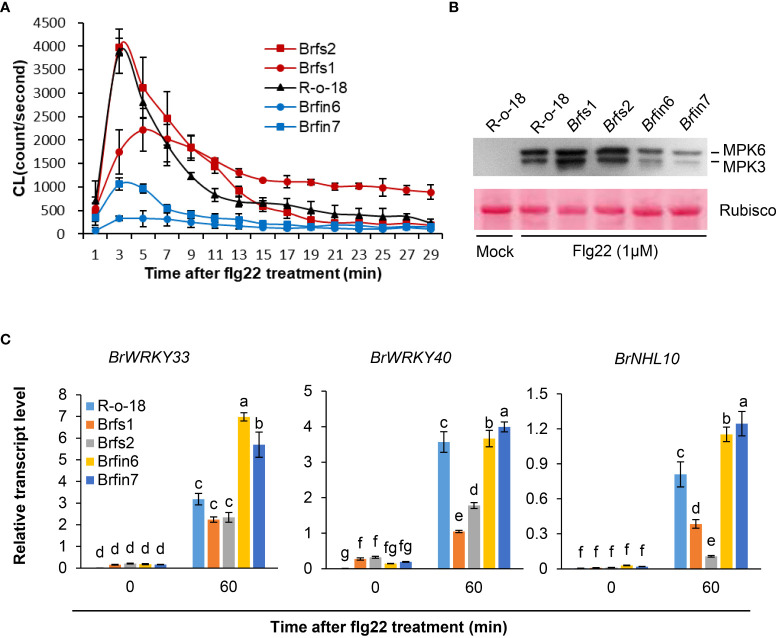
Phenotypes of *Br*fs and *Br*fin lines during flg22-triggered signaling. **(A)** Flg22-induced ROS generation in liquid-grown intact seedlings of *B. rapa* inbred lines treated with 1 µM flg22. Error bars represent standard deviations of 24 independent samples. Similar results were obtained in two independent experiments. **(B)** Dual phosphorylation of the TEY motif in MPK3 and MPK6 in leaf discs. Phosphorylated MAPKs corresponding to MPK3 and MPK6 are indicated. Activated MAPKs were detected by immunoblotting using an antibody against Phospho-p44/42 MAPK (Erk1/2) (Cell Signaling Technology). The experiment was performed three times with similar results. Before transferring proteins to the PVDF membrane, equal protein loading was confirmed by comparing the fluorescence intensity of Rubisco in stain-free gels. **(C)** Transcript levels of PAMP-induced genes in R-o-18, *Br*fs, and *Br*fin seedlings. Twelve-day-old *B. rapa* seedlings were treated with 1 µM flg22 for 60 min, after which *BrWRKY33*, *BrWRKY40*, and *BrNHL10* transcript levels were determined using qRT-PCR. Gene transcript levels were normalized to *ACT2* transcript levels. Error bars represent the standard deviation of three replicates. Similar results were obtained in at least two independent experiments. Different letters indicate significant differences among plant genotypes (α = 0.05, one-way ANOVA and Duncan test; SPSS software).

*Br*fs1 and *Br*fs2 were the most sensitive lines in the flg22-induced SGI assay of 455 *B. rapa* inbred lines, whereas *Br*fin6 and *Br*fin7 were the most insensitive lines. As R-o-18 was moderately sensitive to flg22 (**Figure 1B**), it was selected as the control line in the subsequent flg22 response assays. *Br*fin6, *Br*fin7, *Br*fs1, and *Br*fs2 plants were obtained from the doubled haploid (DH) production system, a protocol used to generate homozygous *B. rapa* plants by culturing microspores isolated from the young flower buds of each F_1_ plant (Broughton et al., 2014). We further analyzed whether these four selected *Br*fs and *Br*fin lines increased or inhibited other flg22-induced responses compared to R-o-18. Since MAPK phosphorylation and ROS production are rapid and transient responses associated with two parallel flg22 signaling pathways, we compared these activities among the selected *Br*fs and *Br*fin lines and R-o-18. We measured the flg22-triggered oxidative burst in selected *B. rapa* accessions using an L-012-based chemiluminescence detection system (Yi et al., 2014). Our assays revealed a substantial decrease in flg22-induced ROS production in *Br*fin 6 and *Br*fin7 (**Figure 3A**). For the *Br*fs1 and *Br*fs2 lines, we expected a high level of flg22-triggered oxidative burst, but similar to that of R-o-18 (**Figure 3A**). To analyze flg22-induced MAPK activation, *B. rapa* seedlings were treated with flg22 or water and analyzed by immunoblotting with total protein extracts and an anti-phospho-p44/p42 antibody that specifically recognizes the phosphorylated forms of MPK3 and MPK6 (Flury et al., 2013). We expected robust flg22-induced MPK3 and MPK6 activation in *Br*fs1 and *Br*fs2 lines, while no clear change in flg22‐induced MAPK activation was observed in *Br*fs lines compared to R-o-18 (**Figure 3B**). However, activation of MPK3 and MPK6 was significantly suppressed in *Br*fin6 and *Br*fin7 (**Figure 3B**). Hypersensitivity of *Br*fs lines to flg22 was observed only in SGI, but *Br*fin lines showed consistent characteristics in almost all tested flg22 responses. The *Br*fin6 and *Brf*in7 lines were insensitive to flg22.

### Expression analysis of PAMP-induced genes (PIGs) in *Br*fs and *Br*fin lines following flg22 treatment

Since MAPK activation is linked to PAMP-induced transcriptional reprogramming (Zipfel et al., 2004; Fiil et al., 2009), we analyzed PAMP-induced gene (PIGs) expression in *B. rapa* seedlings using PTI marker genes. We selected three marker genes, *WRKY33*, *WRKY40*, and *NHL10*, because their expression is highly induced by flg22 within 30 min (Navarro et al., 2004; Zipfel et al., 2004; Boudsocq et al., 2010). Since *Br*fin lines significantly suppressed flg22-induced MPK phosphorylation, we expected that they would have a relatively low level of flg22-induced PIGs expression. However, as shown in **Figure 3C**, PIGs expression levels in *Br*fin lines are higher than in R-o-18. Meanwhile, the expression levels of PIGs in *Br*fs lines were somewhat similar to or significantly lower than in R-o-18 (**Figure 3C**). Thus, these results suggest that flg22-induced ROS production and MAPK activation may not affect the pattern of PIGs expression in *Br*fs and *Br*fin lines. Furthermore, these results imply that there may be other regulatory factors affecting PIGs expression and flg22-triggered immunity in the selected *Br*fs and *Br*fin lines.

### Expression analysis of pathogen-induced genes in *Br*fs and *Br*fin lines following *P. brasiliense* inoculation

**Figure 4:**
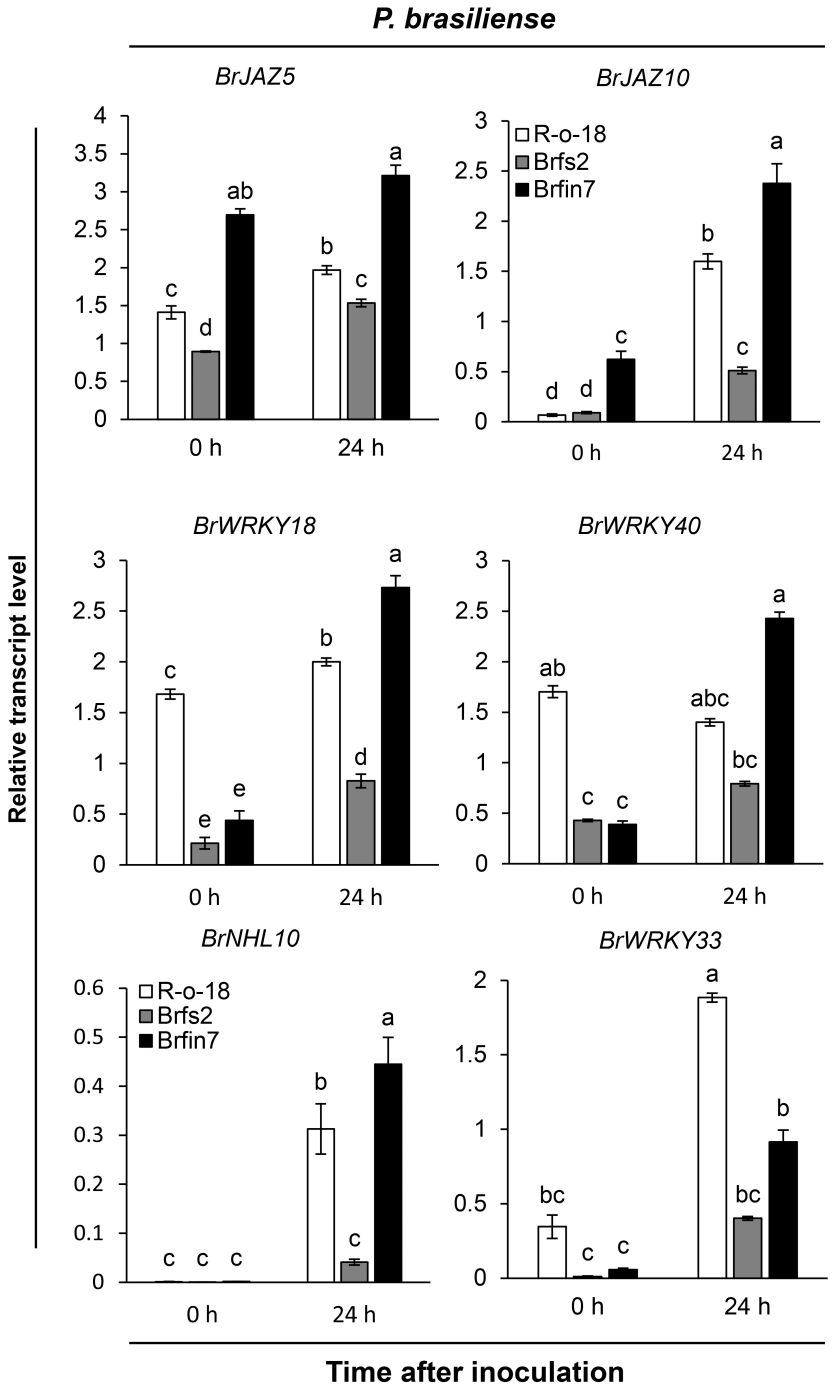
Transcript levels of pathogen-induced genes in R-o-18, *Br*fs2, and *Br*fin7 plants. Four-week-old *B. rapa* plants were inoculated with *P. brasiliense* and sampled at 24 h post-inoculation. The *BrJAZ25*, *BrJAZ10*, *BrWRKY40*, *BrWRKY18*, *BrNHL10* and *BrWRKY33* transcript levels were determined using qRT-PCR. Gene transcript levels were normalized to the *BrACT2* transcript levels. Error bars represent the standard deviation of three replicates. Similar results were obtained in at least two independent experiments. Different letters indicate significant differences among plant genotypes (α = 0.01, one-way ANOVA and Duncan test; SPSS software).

To analyze how altered flg22 responses affect the defense signaling of *Br*fs or *Br*fin lines against *P. brasiliense*, we first compared the expression patterns of several defense marker genes in selected *B. rapa*. *Br*fs2, a line sensitive to flg22 and having the lowest *P. brasiliense*-induced JA accumulation level, and *Br*fin7, a line insensitive to flg22 and having high JA production, were selected to compare the levels of *P. brasiliense*-induced marker gene expression (**Figure 4**). We used Arabidopsis *JAZ5* and *JAZ10*, which are JA- or pathogen-responsive genes (Chung et al., 2008; Demianski et al., 2012; Valenzuela et al., 2016), as JA signaling markers. In the expression analysis of *BrJAZ5* and *BrJAZ10* (**Figure 4**), we observed differences in the expression levels of R-o-18, *Br*fs2, and *Br*fin7 before *P. brasiliense* infection. Interestingly, in the case of *Br*fin7, the expression levels of *BrJAZ5* and *BrJAZ10* were significantly higher than those in the other two lines before *P. brasiliense* infection. In particular, the steady-state expression level of *BrJAZ5* in the *Br*fin7 line before inoculation was prolonged to 24 h after *P. brasiliense* infection. Furthermore, the *Br*fs2 line had the lowest *P. brasiliense*-induced *BrJAZ5*/*BrJAZ10* expression level among the three tested lines (**Figure 4**), which was consistent with the *P. brasiliense*-induced JA level (**Figure 2C**). Arabidopsis *WRKY33* is a key positive regulator of resistance to the necrotrophic fungi *Alternaria brassicicola* and *Botrytis cinerea* (Zheng et al., 2006; Birkenbihl et al., 2012). Arabidopsis *WRKY18* and *WRKY40* act redundantly to negatively regulate resistance to the hemibiotrophic pathogen *Pseudomonas syringae* but positively regulate resistance to *B. cinerea* (Xu et al., 2006). In Arabidopsis, *NHL10* is abundantly expressed in senescing leaves and during the hypersensitive response caused by exposure to an avirulent cucumber mosaic virus (Zheng et al., 2004). Next, we compared the changes in the expression of *BrWRKY33*, *BrWRKY18*, *BrWRKY40*, and *BrNHL10* among R-o18, *Br*fs2, and *Br*fin7 plants before and after *P. brasiliense* inoculation. *P. brasiliense* inoculation significantly suppressed the induced expression of *BrWRKY33*, *BrWRKY18*, *BrWRKY40*, and *BrNHL10* in the *P. brasiliense* susceptible plant, *Br*fs2, compared to *Br*fin7 (**Figure 4**). In response to *P. brasiliense*, the expression levels of *BrWRKY18* and *BrWRKY40* were greater in the resistant plant *Br*fin7 than in R-o-18 at 24 h post-inoculation. These results indicate that the JA signaling pathway plays a crucial role in plant defense against *P. brasiliense*.

### Exogenous JA suppressed the disease development of *B. rapa* infected with *P. brasiliense*

**Figure 5:**
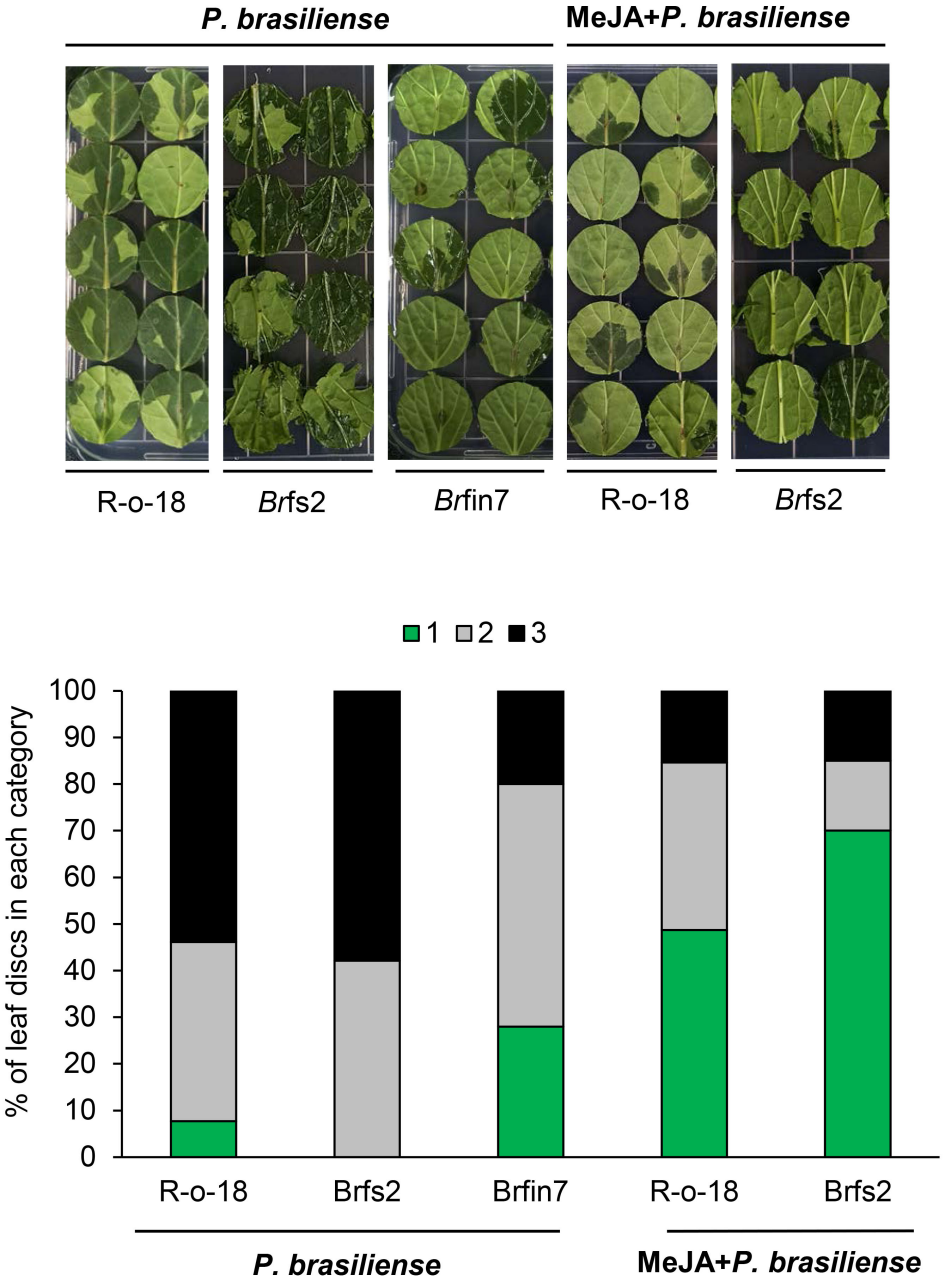
Suppressed necrotic symptom development of MeJA-pretreated *B. rapa* leaves to *P. brasiliense*. Six-week-old *B. rapa* plants were pretreated with MeJA (1 mM) or water and inoculated with 5 μl *P. brasiliense* suspension (1 × 10^8^ CFU/mL) after 24 h. Disease development was scored on the basis of the leaf disc lesion area (0% = 1, 1%–50% = 2, 51%–100% = 3) at 24 h post-inoculation. The bars represent the percentage of leaves with a specific disease severity score (n ≥ 30). The experiment was repeated twice with similar results.

Previous results (**Figures 2**, **5**) showed that the *P. brasiliense*-induced JA level positively affected defense-related gene expression. Line *Br*fin7 exhibited suppressed PTI. However, their *P. brasiliense*-induced defense-related gene expression levels and JA content were higher than that of R-o-18. In contrast, the flg22 sensitive line, *Br*fs2, had significantly lower *P. brasiliense*-induced defense-related gene expression levels and JA accumulation than *Br*fin7. Therefore, we speculated that the resistance of the *Br*fin lines to *P. brasiliense* was associated with considerable accumulation of JA. We investigated whether the application of exogenous JA could decrease the susceptibility of *Br*fs2 to *P. brasiliense*. An earlier investigation demonstrated that the defense response of calla lily to *P. brasiliense* involves the JA/ET signaling pathway (Luzzatto et al., 2007). To evaluate the disease severity of JA-pretreated *B. rapa* inoculated with *P. brasiliense*, leaf lesions were scored according to a modified version of a previously reported method (Luzzatto et al., 2007). Leaf discs were inoculated with *P. brasiliense* 24 h after treatment with MeJA or water. The necrotic area and infection rate were recorded 24 h later (48 h after hormone pretreatment). Application of 1 mM MeJA suppressed disease development (**Figure 5**). Specifically, MeJA pretreatment significantly decreased the soft rot disease development in *Br*fs2 from 100% to 30% (**Figure 5**). Moreover, the disease symptoms on *Br*fs2 leaves pretreated with MeJA were less severe than those on the leaves of *Br*fin7, which were resistant to *P. brasiliense*. These results suggest that activated JA signaling positively affects the resistance to *P. brasiliense* in *B. rapa.*

### *Br*fin7 had higher *P. brasiliense*-induced neoglucobrassicin levels than *Br*fs2

**Figure 6:**
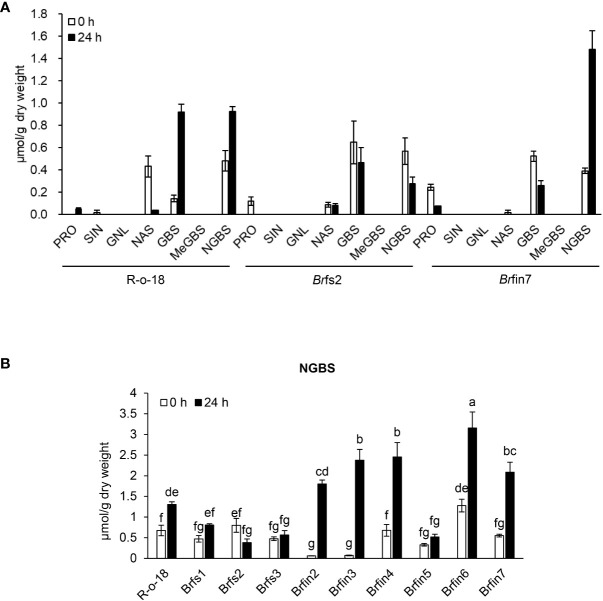
Basal or *P. brasiliense*-induced glucosinolate contents in *B. rapa* leaves. **(A)** Individual glucosinolate content in *B. rapa* before and after *P. brasiliense* inoculation. **(B)** *P. brasiliense*-susceptible lines *Br*fs1, *Br*fs2, and *Br*fs3 produced less *P. brasiliense*-induced NGBS than R-o-18. Individual leaf glucosinolate contents were quantified before the inoculation and at 24 h post-infection. Pro, progoitrin; SIN, sinigrin; GNL, gluconapoleiferin; NAS, gluconasturtiin; GBS, glucobrassicin; MeGBS, 4-methoxyglucobrassicin; NGBS, neoglucobrassicin. Error bars represent standard deviations of three replications. Different letters indicate significant differences among plant genotypes (α = 0.05, one-way ANOVA and Tukey HSD test; SPSS software).

JA is a plant hormone involved in chemical and physiological defense responses. Although JA does not directly affect plant-pathogen interactions, it contributes to an intracellular signaling cascade that induces the production of secondary metabolites that are important for plant defenses (Campos et al., 2014). In this study, activated JA signaling was revealed to be important for restricting the infection of *B. rapa* by *P. brasiliense* (**Figures 5**, **6**). As JA is an elicitor that stimulates IGL biosynthesis (Ku et al., 2014), we analyzed *B. rapa* glucosinolate contents to elucidate the effect of JA accumulation on defense signaling in response to *P. brasiliense*. Individual glucosinolates were analyzed in the R-o-18, *Br*fs2, and *Br*fin7 lines before and after *P. brasiliense* inoculation (**Figure 6A**). As expected, *P. brasiliense* inoculation significantly influenced the IGL contents (**Figure 6A**), and the glucobrassicin (GBS) and neoglucobrassicin (NGBS) contents increased over 2-fold after R-o-18 was inoculated with *P. brasiliense*. However, there were no pathogen-related changes in the content of either aliphatic (progoitrin, sinigrin, and gluconapoleiferin) or aromatic (gluconasturtiin) glucosinolates (**Figure 6A**). Interestingly, the *Br*fin7 line with a high JA biosynthesis level exhibited *P. brasiliense*-induced NGBS accumulation twice as high as that of R-o-18, whereas in *Br*fs2 with a low pathogen-induced JA accumulation level, *P. brasiliense* inoculation did not affect the NGBS level. Our results (**Figures 5** **-** **7**) suggested that *P. brasiliense* resistance observed in *Br*fin lines was strongly correlated with activated JA signaling. We wondered whether other *Br*fin lines that produce relatively high amounts of pathogen-induced JA also have relatively high *P. brasiliense*-induced NGBS levels, similar to *Br*fin7. Thus, we compared NGBS content in three *Br*fs lines, six *Br*fin lines, and R-o-18 24 h after *P. brasiliense* inoculation (**Figure 6B**). Almost all *Br*fin lines (except *Br*fin5) accumulated significantly higher *P. brasiliense*-induced NGBS than the R-o-18 line. In contrast, all *Br*fs lines showed significantly lower *P. brasiliense*-induced NGBS accumulation compared to R-o-18. In the *Br*fs lines, NGBS levels were almost unaffected by *P. brasiliense* inoculation.

**Figure 7:**
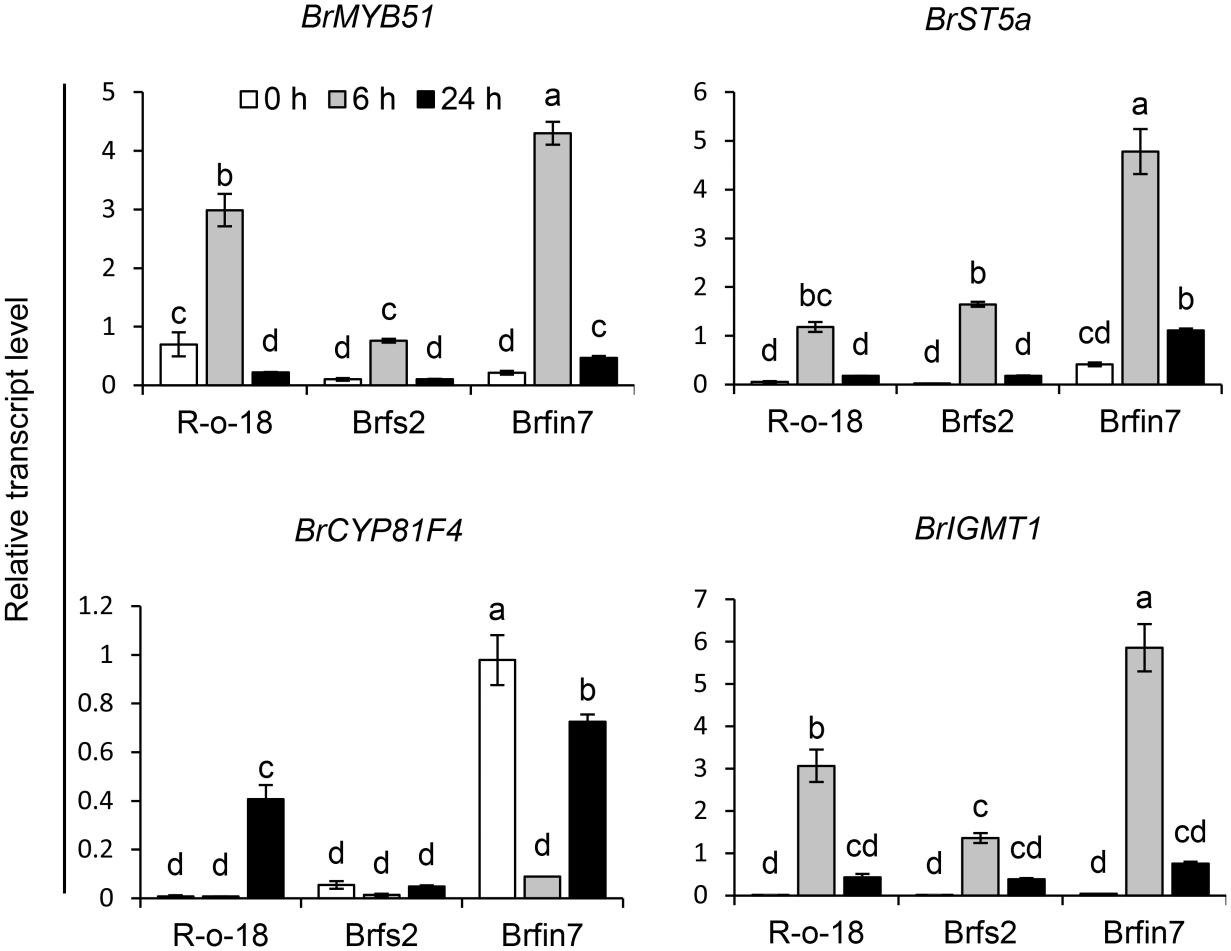
Analysis of the expression patterns of indole glucosinolate biosynthesis genes in *B. rapa* after the *P. brasiliense* inoculation. Transcript levels of indole glucosinolate biosynthesis genes in R-o-18, *Br*fs2, and *Br*fin7 plants. Four-week-old *B. rapa* plants were inoculated with *P. brasiliense* and sampled at the indicated post-infection time-points. The *BrMYP51*, *BrST5a*, *BrCYP81F4*, and *BrIGMT1* transcript levels were determined by qRT-PCR. Gene transcript levels were normalized against the *ACT2* transcript level. Error bars represent standard deviations of three replications. Similar results were obtained in at least two independent experiments. Different letters indicate significant differences among plant genotypes (α = 0.01, one-way ANOVA and Tukey HSD test; SPSS software).

### *Br*fin7 accumulates more pathogen-induced glucosinolate biosynthesis genes than *Br*fs2

The upregulated expression of glucosinolate biosynthesis genes is associated with an increased abundance of individual glucosinolates in plants (Robin et al., 2016; Yi et al., 2016; Robin et al., 2017a; Robin et al., 2017b). Thus, we analyzed the correlations among the IGL profiles of the R-o-18, *Br*fs2, and *Br*fin7 lines and the expression of IGL biosynthesis genes following infection with *P. brasiliense*. MYB transcription factor MYB51 regulates IGL biosynthesis (Celenza et al., 2005; Gigolashvili et al., 2008; Malitsky et al., 2008). Arabidopsis SULFOTRANSFERASE 5A (ST5a) encodes a desulfoglucosinolate sulfotransferase that is involved in the final step of glucosinolate core structure biosynthesis (Klein et al., 2006). In Arabidopsis, CYP81F4 catalyzes the conversion of I3M to 1OH-I3M, which in turn is converted to 1MO-I3M (NGBS) by indole glucosinolate methyltransferase 1 (IGMT1) or IGMT2 (Pfalz et al., 2009; Pfalz et al., 2011). *P. brasiliense*-induced gene expression in R-o-18, *Br*fs2, and *Br*fin7 leaf tissues were analyzed at 0, 6, and 24 h after infection. Since a previous report demonstrated that the most critical defense regulation period against *P. brasiliense* in Chinese cabbage was from 6–12 h after infection (Liu et al., 2019), we included an early time point, 6 h after infection. Expression of IGL biosynthesis genes was detected at different time points after *P. brasiliense* infection. The results showed that *BrMYB51*, *BrST5a*, and *BrIGMT1* expression was strongly induced by *P. brasiliense* infection in R-o-18 at 6 h after infection and then decreased at 24 h after infection. In contrast, *BrCYP81F4* was strongly induced in R-o-18 at 24 h post-inoculation. For *BrMYB51* and *BrIGMT1*, a distinct difference in *P. brasiliense*-induced expression was observed between R-o-18, *Br*fs2, and *Br*fin7 6 h after *P. brasiliense* inoculation; whereas in the case of *BrCYP81F4*, features between lines were observed 24 h after *P. brasiliense* inoculation. Taken together, expression analysis revealed that the upregulation of key biosynthetic genes of IGL (*BrMYB51*, *BrCYP81F4*, and *BrIGMT1*) was significantly compromised in *Br*fs2 compared with that in R-o-18 after inoculation with *P. brasiliense* (**Figure 7**). Meanwhile, the *P. brasiliense* response expression of IGL biosynthesis genes in *Br*fin7 was significantly higher than that in R-o-18. These results suggested that higher IGL biosynthesis gene expression levels may be related to a more significant accumulation of NGBS in *Br*fin7 than in *Br*fs2.

## Discussion

### Genetic requirements for individual PAMP responses may differ among *B. rapa* inbred lines

*P. brasiliense* causes the destructive soft rot disease in many economically important vegetables, including *B. rapa*. However, little is known about the mechanism underlying this molecular battle between plant immunity and *P. brasiliense* virulence. In this study, we analyzed whether flg22-induced immunity affects the development of the soft rot disease following infection with *P. brasiliense*. Flagellin perception restricts bacterial infection and contributes to plant disease resistance. Arabidopsis ecotype Ws-0 rapidly develops severe disease symptoms after being sprayed with *Pst* DC3000 because of a natural deficiency in flagellin perception (Zipfel et al., 2004). Under natural conditions, the hemibiotrophic bacterial pathogen *Pst* DC3000 enters host plants through wounds or natural openings (e.g., stomata) and then multiplies, resulting in high population densities in intercellular spaces (Beattie and Lindow, 1995). In contrast, *P. brasiliense* is an aggressive necrotrophic bacterium that produces PCWDEs as its primary virulence determinant. Therefore, it is necessary to analyze whether flg22-triggered immunity positively affects the resistance of plants to *P. brasiliense*, as in the *Pst* DC3000 and Arabidopsis interaction. Chinese cabbage (*Brassica rapa* subsp. *pekinensis*) is the most widely grown vegetable crop in Asia. Therefore, there is a substantial abundance of genetic and genomic resources available for the improvement of Brassica crops. Furthermore, fundamental research on Arabidopsis may apply to *B. rapa* because both species belong to the family Brassicaceae. Previous research revealed ~ 80% amino acid sequence identity and 90% amino acid sequence similarity between the *Brassica* and Arabidopsis FLS2 LRR domains as well as the functionality of the LRR domains of *Brassica* FLS2 homologs in Arabidopsis (Dunning et al., 2007). This high degree of conservation is indicative of the importance of this receptor for *B. rapa* defense against pathogens (Kim et al., 2020). To clarify the defense-related signaling in *B. rapa* induced by *P. brasiliense*, we conducted a forward genetics screening to isolate *B. rapa* inbred lines exhibiting impaired flg22-induced SGI (**Figure 1**). Of the 455 *B. rapa* lines screened, three flagellin-sensitive (*Br*fs) lines and six flagellin-insensitive (*Br*fin) lines had reproducibly significant alterations in their flg22-induced responses compared to R-o-18 (**Figure 2A**).

We focused on *Br*fin6 and *Br*fin7, in which almost all the examined flg22-triggered responses were severely suppressed, with the exception of PIG expression (**Figure 3C**). Flg22-induced rapid activation of MAPK cascades is one of the critical components that regulate transcriptional changes in elicited cells. For example, in Arabidopsis protoplasts, MPK6 and MPK3 are phosphorylated upon flg22 treatment and activate WRKY transcription factors (TFs) (Nuhse et al., 2000; Asai et al., 2002). Fifteen WRKY TF genes were strongly induced 30 min after flg22 treatment in Arabidopsis seedlings including *WRKY18*, *WRKY33*, and *WRKY40* (Zipfel et al., 2004). In the current study, we also observed strong expression of *WRKY33* and *WRKY40* together with MAPK activation, following flg22 elicitation in R-o-18 (**Figure 3**). Interestingly, there was no correlation between the level of MAPK activation and the transcript levels of WRKY TF genes in *Br*fs and *Br*fin lines (**Figure 3C**). These results suggest that to regulate PIGs induction, another signaling component is required in addition to the phosphorylation of MPK3/MPK6 in *B. rapa*. We also analyzed the early flg22 responses of *Br*fs1 and *Br*fs2, which were selected as the lines most sensitive to flg22 in the SGI assay, along with the *Br*fin lines. Interestingly, the level of flg22-triggered oxidative burst and phosphorylation of MAPKs was similar to that of R-o-18, whereas the expression level of PIGs was significantly suppressed compared to that of R-o-18 (**Figure 3**). Previous reports have suggested that genetic requirements vary among individual PAMP responses. For example, ethylene sensing is required for flg22-induced ROS production and callose deposition but not for flg22-triggered MAP kinase activation, seedling growth arrest, and induced resistance (Zipfel et al., 2004; Adams-Phillips et al., 2008; Clay et al., 2009; Mersmann et al., 2010). Therefore, although *Br*fin6 and *Br*fin7 were minimally responsive to flg22, their PIGs expression levels were relatively high. In an earlier investigation involving Arabidopsis, flg22-induced callose deposition was undetectable in *cyp81F2-1* and *cyp81F2-2* mutants, in which the PIG *CYP81F2* had been mutated (Clay et al., 2009). In the current study, compared with R-o-18, flg22-induced *BrCYP81F2* expression was lower and callose deposition was suppressed in *Br*fin7 (**Supplementary Figure S2**). In contrast, we observed strong flg22-induced *BrCYP81F2* expression and callose deposition in *Br*fin6 cells (**Supplementary Figure S2**). Hence, some of the examined flg22-induced responses varied between *Br*fin6 and *Br*fin7, implying that the genetic requirements for individual PAMP responses differed between the two *Br*fin lines.

### Relationship between phytohormones and early flg22 responses in *B. rapa*

Previous studies have indicated that PAMP-induced responses are independent of phytohormone signaling (Zipfel et al., 2004; Ferrari et al., 2007). However, the oxidative burst is reportedly diminished in ethylene-insensitive mutants (Mersmann et al., 2010). Our previous report also showed that a clear increase in ROS production was detected in *fad7/fad8, coi1*, and *jar1* mutants, which have impaired JA biosynthesis and signaling (Yi et al., 2014). Overall, ET signaling had a positive effect on flg22-triggered oxidative burst, but JA signaling tended to inhibit ROS production. Additionally, PAMPs have been reported to stimulate JA and ethylene (ET) production (Doares et al., 1995; Simpson et al., 1998; Kunze et al., 2004), as well as upregulate genes encoding proteins involved in the biosynthesis of JA and ET (Moscatiello et al., 2006) or pathogenesis-related proteins linked to SA-mediated responses (Gomez-Gomez et al., 1999). In the present study, we analyzed the levels of specific phytohormones (ABA, SA, and JA) in *B. rapa* before and after *P. brasiliense* inoculation and revealed that the JA level was clearly higher in the *Br*fin lines than in *Br*fs lines (**Figure 2C**). Although the endogenous basal level of JA in the *Br*fin lines was not higher than that in the *Br*fs lines, five *Br*fin lines had high *P. brasiliense*-induced JA levels, which may be involved in the fast and strong activation of the JA-dependent signaling pathway in *Br*fin lines (**Figure 2C**). These results may correlate with the highly suppressed flg22-triggered oxidative burst in *Br*fin6 and *Br*fin7 lines, which accumulate relatively high JA (**Figure 3A**). Consistent with our results, a study by Denoux et al. (Denoux et al., 2008) showed that treatment with flg22 triggers a fast response in the early stages of multiple defense signaling pathways mediated by SA, JA, and ET. Early responses are associated with JA, and late responses are mediated mainly by SA. In addition, ET biosynthesis has been detected in flg22-treated Arabidopsis (Denoux et al., 2008). These results suggest that in addition to defense responses that are activated independently of defense hormone signaling, activated plant hormone signaling may also stimulate flg22 responses.

In Arabidopsis, the NADPH oxidase responsible for the PAMP-triggered oxidative burst is the plasma membrane-localized RBOHD (Nuhse et al., 2007; Zhang et al., 2007). The Arabidopsis *rbohD* mutant shows impaired PAMP-induced ROS burst and stomatal closure (Macho et al., 2012; Marino et al., 2012). The extent of flg22-induced activation of MPK3, MPK4, and MPK6 was similar to that in the *rbohd* mutant, suggesting that the ROS burst is not required for MAPK activation (Zhang et al., 2007; Xu et al., 2014). Furthermore, another study confirmed that flg22-induced MPK3/MPK6 activation is similar between wild-type plants and *dde2 ein2 pad4 sid2* quadruple mutants, implying that MAPK activation occurs independently of the SA, JA, and ET signaling pathways (Tsuda et al., 2008). However, Mine et al. (Mine et al., 2017) reported that ABA and JA mediate inactivation of the immune-associated MAP kinases, MPK3 and MPK6, in *Arabidopsis thaliana*. In our study, *Br*fin lines had relatively high *P. brasiliense*-induced JA or ABA (**Figure 2C** and **Supplementary Figure S1**) compared to R-o-18, and they represented suppressed flg22-induced MAPK activation compared to R-o-18 (**Supplementary Figure S3**). These results are consistent with those described in a published report (Mine et al., 2017) and may help explain the traits in the *Br*fin lines related to suppressed flg22 responses (**Supplementary Figure S3** and **Figure 3B**).

### Relationships between activated JA signaling and defense-related gene expression by *P. brasiliense*

In Arabidopsis, although *WRKY33*, *WRKY40*, and *NHL10* are early flg22 response genes (Navarro et al., 2004; Zipfel et al., 2004), several studies have shown that pathogens also induce the expression of these genes (Zheng et al., 2004; Xu et al., 2006; Zheng et al., 2006; Birkenbihl et al., 2012). WRKY40 and WRKY33 transcription factors modulate the SA and JA pathways and function as activators of JA-dependent defense pathways and repressors of SA signaling (Xu et al., 2006; Zheng et al., 2006). Arabidopsis WRKY33 is a key transcriptional regulator of hormonal and metabolic activities that protect plants from *B. cinerea* strain 2100 (Birkenbihl et al., 2012). During this plant-pathogen interaction, WRKY33 positively regulates the expression of target genes involved in camalexin biosynthesis as well as JA/ET-related downstream signaling, while negatively regulating ABA-dependent signaling (Liu et al., 2015). As *P. brasiliense* is also a necrotrophic pathogen like *B. cinerea*, we predicted that the *WRKY33* and *WRKY40* genes have similar expression patterns in response to *P. brasiliense*. However, following *P. brasiliense* inoculation, *BrWRKY33* expression levels were lower in the *P. brasiliense*-resistant line *Br*fin7 than in R-o-18, whereas *Br*WRKY40 was more highly expressed in the *Br*fin7 line than in R-o-18 (**Figure 4**). Therefore, the lower *P. brasiliense*-induced *BrWRKY33* expression levels in *Br*fin7 than in R-o-18 imply that although both *B. cinerea* and *P. brasiliense* are necrotrophic pathogens, their infection of host plants involves different hormonal and metabolic processes. A previous report also revealed that different *B. cinerea* strains employ diverse strategies to invade and colonize plant hosts (Derckel et al., 1999; Kliebenstein et al., 2005). During infection with *B. cinerea* strain B05.10, WRKY33-mediated host defenses are suppressed by the pathogen. Ectopic expression of *WRKY33* leads to elevated ABA levels and results in plants that are completely resistant to *B. cinerea* strain B05.10 (Liu et al., 2017).

### Relationships between activated JA signaling and defense response to *P. brasiliense*

JA signaling mediates plant defenses against necrotrophic pathogens, including bacteria (e.g., *Pectobacterium atrosepticum*), fungi (e.g., *A. brassicicola*, *B. cinerea*, *Plectosphaerella cucumerina*, and *Fusarium oxysporum*), and oomycetes (e.g., *Pythium* spp.) (Campos et al., 2014; Yan and Xie, 2015). The *P. brasiliense*-induced JA content in *Br*fin lines was relatively higher than that in R-o-18 (**Figure 2C**). Accordingly, we hypothesized that changes in the regulation of phytohormones might affect the development of necrotrophic bacterial diseases in *br*fin lines. *Br*fin6 and *Br*fin7 were relatively resistant to *P. brasiliense* (**Figure 2**). This result suggests that relatively high *P. brasiliense*-induced JA levels may contribute to reduced necrotic symptom development (**Figure 2**). Furthermore, MeJA pre-treatment suppressed the development of necrotic symptoms by *P. brasiliense*, and these results support our hypothesis (**Figure 5**). The *P. brasiliense* susceptible line R-o-18 showed that more than 80% of the tested leaf discs were necrotic. However, MeJA pretreatment decreased the number of necrotic leaf discs from more than 80% to 50%, reflecting the inhibitory effect of MeJA on the development of the soft rot disease (**Figure 5**). Furthermore, MeJA pretreatment effectively inhibited necrosis development in the *P. brasiliense* hyper-susceptible line, *Br*fs2, and as a result, represented stronger resistance to *P. brasiliense* than *Br*fin7, the resistant line (**Figure 5**). In other published reports, exogenous JA and MeJA treatments have been used to induce plant responses, resulting in pathogen resistance. For example, an investigation of the interaction between Arabidopsis and *P. brasiliense* revealed that MeJA stimulates IGL accumulation (Brader et al., 2001). Specifically, experiments involving plant hormone signaling-deficient mutants have indicated that JA, but not SA or ET, mediates pathogen-induced IGL biosynthesis. *P. brasiliense*-induced IGL accumulation was not observed in the JA-insensitive mutant *coi1-1*. However, similar to wild-type plants, *NahG* and ethylene-insensitive *ein2-1* mutant plants reportedly lack changes in IGL levels and MeJA- or elicitor-induced IGL accumulation (Brader et al., 2001). In this study, inoculation of R-o-18 plants with *P. brasiliense* increased GBS content by more than 6-fold. Interestingly, the *Br*fin lines with high *P. brasiliense*-induced JA levels had GBS contents that were half that of plants before inoculation. However, *P. brasiliense*-induced NGBS levels were almost 2-fold higher in *Br*fin lines than in R-o-18. These results suggest that NGBS may be more important than other *P. brasiliense*-inducible IGLs for the *P. brasiliense* resistance-related signaling pathway in *B. rapa* (**Figure 6**). We also analyzed the correlation between *P. brasiliense*-induced NGBS accumulation and IGL biosynthesis gene expression patterns. In response to *P. brasiliense* inoculation, all examined genes (*BrMYB51*, *BrST5a*, *BrCYP81F4*, and *BrIGMT1*) were more highly expressed in *Br*fin7 than in R-o-18 (**Figure 7**). MYB transcription factors MYB34, MYB51, and MYB122 regulate IGL biosynthesis. Dominant mutants or lines overexpressing the genes encoding these transcription factors have increased IGL content and upregulated IGL biosynthesis gene expression levels, whereas the corresponding loss-of-function mutants have decreased IGL content and downregulated IGL biosynthesis gene expression levels (Celenza et al., 2005; Gigolashvili et al., 2008; Malitsky et al., 2008). In this study, we analyzed the *P. brasiliense*-induced expression pattern of *BrMYB51* and found that the expression level was significantly higher than that of R-o-18 at either 6 or 24 h of inoculation. In Arabidopsis, CYP81F4 belongs to a small cytochrome P450 monooxygenase family, which also includes CYP81F1, CYP81F2, and CYP81F3. CYP81F4 catalyzes the conversion of I3M to 1OH-I3M, which in turn is converted to 1MO-I3M (NGBS) by either indole glucosinolate methyltransferase 1 (IGMT1) or IGMT2 (Pfalz et al., 2009; Pfalz et al., 2011). Our expression analysis showed that the *P. brasiliense*-induced expression levels of *BrIGMT1*, a closely related gene involved in NGBS biosynthesis, were higher in *Br*fin7 than in R-o-18 6 h after infection (**Figure 7**). In the case of *BrCYP81F4*, the *P. brasiliense* response expression pattern differed from that of the other tested genes. In the control line R-o-18, the *P. brasiliense*-induced expression level was strongly increased after 24 h instead of 6 h, and the expression level of *BrCYP81F4* in *Br*fin7 was significantly higher than R-o-18 in this time. Taken together, these results indicate that the *P. brasiliense*-induced expression levels of key biosynthetic genes of IGLs, which are closely related genes involved in NGBS biosynthesis, were higher in the *Br*fin lines than in R-o-18, which might correlate with high NGBS accumulation following *P. brasiliense* infection in *Br*fin7 (**Figure 7**). The *P. brasiliense* hyper-susceptible line *Br*fs2 had the lowest *P. brasiliense*-induced JA and NGBS levels among the tested plant lines (**Figures 2C**, **7B**). In addition, the induced expression of IGL biosynthesis genes after *P. brasiliense* inoculation of *Br*fs2 was very low, within 20% of that of *Br*fin7 (**Figure 7**). As expected, *P. brasiliense*-induced NGBS accumulation in *Br*fs2 was very low, unaffected by *P. brasiliense* infection, or decreased slightly (**Figure 6**). These findings suggest a correlation between JA-mediated NGBS accumulation and *P. brasiliense* resistance in *B. rapa.*

## Materials and methods

### Plant growth

*Brassica rapa* plants were grown in soil in a growth chamber at 23°C with a 16-h light/8-h dark photoperiod (i.e., long-day conditions) for 4 weeks. The third leaf from each plant was used for pathogen inoculation tests. To grow *B. rapa* seedlings on Murashige and Skoog (MS) medium, the seeds were surface-sterilized in 25% NaOCl solution for 2 min, washed thrice in sterile water, and sown on MS agar medium containing 3% sucrose. Seedlings were grown in a growth chamber at 23°C, 60% relative humidity, and long-day conditions under white light (140 μmol m^−2^ s^−1^).

### Growth assay

Seedlings grown for five days on MS agar medium in plates were transferred to liquid MS medium supplemented with 10 µM flg22 (three seedlings per 6 mL medium in the wells of 6-well plates) or distilled water. The seedlings were photographed and weighed (i.e., fresh weight) after six days to determine the effect of flg22 treatment on seedling growth.

### Pathogen infection assays

Because the taxonomy of Pectobacterium Genus updated (Portier et al., 2020) we confirmed the taxonomic status of KACC10225 (http://genebank.rda.go.kr/eng/uat/uia/actionMain.do), used in this study as a plant necrotrophic bacterial pathogen. The housekeeping genes *dnaX*, *leuS* and *recA* of KACC10225 were amplified and sequenced. PCR protocols and primers were described in Portier et al. (Portier et al., 2019). The GenBank accession numbers for *dnaX*, *leuS*, and *recA* genes of KACC10225 are OP328786, OP328785, and OP328784 respectively. Because three genes showed ~99% nucleotide sequence identity to those of *Pectobacterium brasiliense* strain BC1 (GenBank accession number: CP009769) we described KACC10225 as *Pectobacterium brasiliense* in this study. To induce *P. brasiliense* systemic infection, *B. rapa* plants were inoculated as previously described (Lee et al., 2020). Briefly, *P. brasiliense* isolate KACC 10225 was cultured in NB broth (Becton, Dickinson, and Co.) for 36 h in an incubator set at 30°C with continuous shaking (200 rpm). The bacterial culture was diluted in distilled water to an OD_600_ of 0.1 (1 × 10^6^ CFU/mL) and then 24-day-old seedlings were inoculated with *P. brasiliense* KACC 10225 by drenching the proximal plant parts with the bacterial suspension. The inoculated plants were incubated in a dew chamber at 25°C for 24 h and then transferred to a growth room set at 25°C and 80% relative humidity with a 12-h photoperiod. Seven days after inoculation, the disease severity was assessed using the following scale: 0 (no symptoms), 1 (chlorosis or 1%–25% rotted), 2 (chlorosis or 25%–50% rotted), 3 (chlorosis or 50%–75% rotted), and 4 (chlorosis or 75%–100% rotted; the plant was dead), which was later converted to a percentage (Lee et al., 2020).

To analyze localized *P. brasiliense* infections, 4-week-old *B. rapa* plants were inoculated, as previously described (Liu et al., 2019). Briefly, the third leaf collected from each plant was cut into 2.3 cm diameter discs using a cork borer and placed on two layers of moistened filter paper in covered square Petri dishes (12.5 × 12.5 cm) to maintain high humidity. The leaf discs were then pierced at the center with a sterile tip, inoculated with 5 µL *P. brasiliense* suspension (OD_600 _= 1), and placed in an incubator (28°C and 90% humidity). We analyzed the symptoms of leaf discs 24 h post-inoculation.

To analyze the effect of JA on the development of the soft rot disease, 4-week-old *B. rapa* plants were inoculated as previously described (Luzzatto et al., 2007; Liu et al., 2019). A 1-mM methyl jasmonate (MeJA; Sigma-Aldrich) solution was applied as a foliar spray 24 h before inoculation. Leaf discs were prepared and inoculated with *P. brasiliense* as described above. Disease symptoms were recorded 24 h later (48 h after MeJA treatment).

### Transcript profiling

For quantitative reverse transcription PCR (qRT-PCR) analysis, 8-day-old seedlings grown under sterile conditions were treated with 1 µM flg22 or H_2_O for 30 min. Four-week-old *B. rapa* plants grown in soil were inoculated with a *P. brasiliense* bacterial solution (OD_600_ = 0.1) and sampled at 0, 6, and 24 h post-inoculation. Total RNA was extracted, and residual DNA was digested using the RNeasy Plant Mini Kit and RNase-Free DNase Set (Qiagen, Hilden, Germany). Purified RNA (1 µg) served as the template for synthesizing cDNA using the ReverTra Ace-α kit with oligo-(dT) primers (Toyobo, Osaka, Japan). qRT-PCR analysis was performed using the TB Green Prefix Ex Taq premix (Takara Bio, Shiga, Japan) and CFX96 Real-Time PCR system (Bio-Rad). The qRT-PCR primers used are listed in **Supplementary Table S1**.

### Reactive oxygen species measurements

Leaf discs prepared from 12-day-old *B. rapa* plants were used to analyze ROS content as previously described (Yi et al., 2014), with minor modifications. Briefly, leaf discs were incubated in a 96-well microplate containing liquid MS medium supplemented with 0.1% (w/v) sucrose. An EnVision 2101 multi-label plate reader (Perkin Elmer, Waltham, MA, USA) was used to measure the L-012-derived chemiluminescence (counts per second) at an emission wavelength of 590 nm.

### MAPK phosphorylation assay and protein detection

The MAPK activity of crude protein extracts from the cotyledons of 8-day-old seedlings treated with 1 µM flg22 for 1 h was determined as previously described (Flury et al., 2013). Specifically, the proteins from the crude extracts were separated by 10% SDS-PAGE and then transferred to a PVDF membrane (Bio-Rad; www.bio-rad.com) using a Mini-Protean II semi-dry electroblotting system (Bio-Rad). Activated MAPKs were detected following a 1 h incubation with Phospho-p44/42 MAPK (Erk1/2) rabbit monoclonal antibody (mAb) (1:2,000; Cell Signaling Technology, www.cellsignal.com) and a subsequent 1 h incubation with anti-rabbit-HRP secondary antibodies (Bio-Rad). The signals were visualized using a Clarity™ Western ECL system (Bio-Rad).

### Aniline blue staining, microscopic analysis, and callose quantification

Cotyledons from 8-day-old *B. rapa* seedlings were treated with 1 µM flg22 or H_2_O; subsequently, they were treated with 95% ethanol, and stained with aniline blue as previously described (Gomez-Gomez et al., 1999) with minor modifications. Briefly, the cotyledons were incubated for at least 24 h in 95%–100% ethanol until the tissues were transparent. They were then washed with 0.07 M phosphate buffer (pH 9) and incubated for 1–2 h in 0.07 M phosphate buffer containing 0.01% (w/v) aniline blue (Sigma). At least ten cotyledons per condition per experiment were examined under ultraviolet light using a TE 2000 epifluorescence microscope (Nikon, Tokyo, Japan). The callose content was quantified using Photoshop CS6 software (Luna et al., 2011) to analyze digital photographs based on the number of white pixels (callose intensity) or the number of callose deposits relative to the total number of pixels covering the plant material.

### Analyses of ABA, JA, and SA contents

Fresh *B. rapa* leaves were harvested at 0 and 24 h post-inoculation, weighed, immediately frozen in liquid nitrogen, and stored at −80 °C. Samples were prepared and plant hormones were analyzed as previously described (Park et al., 2022). Briefly, to extract phytohormones, we used ethyl acetate (HPLC grade, Sigma-Aldrich, Saint Louis, MO, USA) spiked with labeled phytohormones as internal standards: D_6_-ABA, D_4_-SA, and D_6_ -JA in 20 ng μL^−1^. Two steel balls of 3 mm diameter and 1 mL of spiked ethyl acetate were added to 100 mg of ground *B. rapa* leaf sample and vortexed using a Tissuelyser II (Qiagen, Hilden, Germany) for 2 min. Samples were centrifuged at 13,000 rpm and 4°C for 20 min, and supernatants were transferred to a new 2 mL tube each. This extraction process was repeated by adding ethyl acetate (0.5 mL) without an internal standard. Supernatants were evaporated at 30°C using a HyperVAC-MAX (Hanil Scientific Inc., Daejeon, Republic of Korea) until dry. Subsequently, 500 μL 70% methanol (HPLC grade, Sigma-Aldrich) was added to dissolve the dried pellet. After centrifugation at 13, 000 rpm for 10 min at 4°C, 400 μL of the dissolved sample was transferred into an HPLC vial. The extracts were analyzed using an ultra-high-performance liquid chromatography-triple quadrupole mass spectrometer (LCMS-8050, Shimadzu Corp., Kyoto, Japan), according to the methods of (Schafer et al., 2016), with some modifications (Joo et al., 2021). Then, 2 μL of the extracts were injected into a C_18_ column (UPLC BEH, 1.7 μm particle size, 100 mm length × 2.1 μm inner diameter, Waters, Milford, MA, USA) and separated using an HPLC system (Shimadzu Corp., Japan). Solvent A consisted of deionized water containing 0.1% (*v*/v) acetonitrile and 0.5% formic acid, whereas solvent B consisted of 100% methanol.

### Analyses of glucosinolate

Samples were prepared and glucosinolates were analyzed as previously described (Park et al., 2019). Glucosinolates were extracted from 100 mg freeze-dried *B. rapa* samples. Briefly, crude glucosinolates from 100 mg of freeze-dried powder were extracted with 1.5 ml of boiling 70% (v/v) MeOH at 70°C for 5 min in a water bath. After centrifugation at 12,000 rpm at 4°C for 10 min, the supernatant was collected into a 5 mL test tube, and the residue was re-extracted twice as described above. The combined supernatants were used as the crude glucosinolate extracts. Crude glucosinolate extracts were passed through a mini-column containing diethylaminoethanol (DEAE) Sephadex A-25 (GE Healthcare, Uppsala, Sweden). For desulfation, the eluates were mixed with 75 µL of purified arylsulfatase and incubated overnight at 25°C. The samples were eluted using 0.5 mL (3× volume) ultrapure water and collected in clean 2.0-mL microcentrifuge tubes. The solutions were filtered using 0.22-µm PTFE syringe filters (Sterlitech Corp., Kent, WA, USA), and the filtrates were collected in amber glass screw thread vials (Thermo Fisher Scientific, USA). The glucosinolates were separated using a reversed-phase Inertsil ODS-3 column (150 × 3.0 mm, 3 μm) and an E-type cartridge guard column (10 × 2.0 mm, 5 μm) at 40°C. Chromatographic separation was performed using a 1200 series HPLC system (Agilent Technologies, Palo Alto, CA, USA) at 227 nm wavelength. The mobile phase consisted of ultrapure water (solvent A) and acetonitrile (solvent B), at a flow rate of 1 mL/min. The gradient program was as follows (40 min): 7%–24% solvent B, 18 min; 24% solvent B, 14 min; 7% solvent B, 32.1 min; and 7% solvent B, 8 min. Individual glucosinolates were identified according to their HPLC peak area ratios and quantified based on their retention times, peak areas, and response factors. Desulfosinigrin (Sigma-Aldrich Co. Ltd., St. Louis, MO, USA) was used as an external reference standard.

### Statistical analysis

All data are presented as the mean ± standard deviation. Transcript abundance, fresh weight, number of callose deposits, symptom development rate, phytohormone content, and individual glucosinolate contents were obtained from at least three independent biological replicates. Analysis of variance (ANOVA) was performed using IBM SPSS Statistics (version 26). One-way ANOVA followed by Tukey’s honestly significant difference (HSD) test or Duncan test was conducted to compare three or more groups.

### Accession numbers

The sequence data included in this study are available from the Brassicaceae Database (BRAD) (http://brassicadb.cn) under the following accession numbers: *BrACT2* (Bra022356), *BrWRKY33* (Bra005104), *BrWRKY40* (Bra035148), *BrNHL10* (Bra017272), *BrMYB51* (Bra016553), *BrST5a* (Bra008132), *BrCYP81F4* (Bra010598), and *BrIGMT1* (Bra012270).

## Data availability statement

The data supporting the findings of this study are available from the corresponding author upon reasonable request.

## Author contributions

Conceptualization, SY. Data curation, SY. Funding acquisition, SY and S-GK. Investigation, ML, SP, LL and GL. Methodology, SY and S-GK. Project administration, SY. Resources, YL. Visualization, SY. Writing the manuscript, SY and S-YK. All authors have read and agreed to the published version of the manuscript.

## Funding

This work was supported by the Ministry of Education of the Republic of Korea and the National Research Foundation of Korea (NRF-2016R1A2B4013170 and NRF-2018K1A3A7A03089858). This research was also funded by the Rural Development Administration (PJ01481603).

## Acknowledgments

The authors are grateful to Dr. Gyung Ja Choi of the Korea Research Institute of Chemical Technology (KRICT) for her helpful discussions and the technical advice on plant pathogenicity assay.

## Conflict of interest

The authors declare that the research was conducted in the absence of any commercial or financial relationships that could be construed as a potential conflict of interest.

## Publisher’s note

All claims expressed in this article are solely those of the authors and do not necessarily represent those of their affiliated organizations, or those of the publisher, the editors and the reviewers. Any product that may be evaluated in this article, or claim that may be made by its manufacturer, is not guaranteed or endorsed by the publisher.
