# Supplementary figures and images for: Jasmonate regulates plant resistance to Pectobacterium brasiliense by inducing indole glucosinolate biosynthesis
Source: Front Plant Sci. 2022 Sep 29;13:964092. doi: 10.3389/fpls.2022.964092 (PMC9559233; doi:10.3389/fpls.2022.964092)

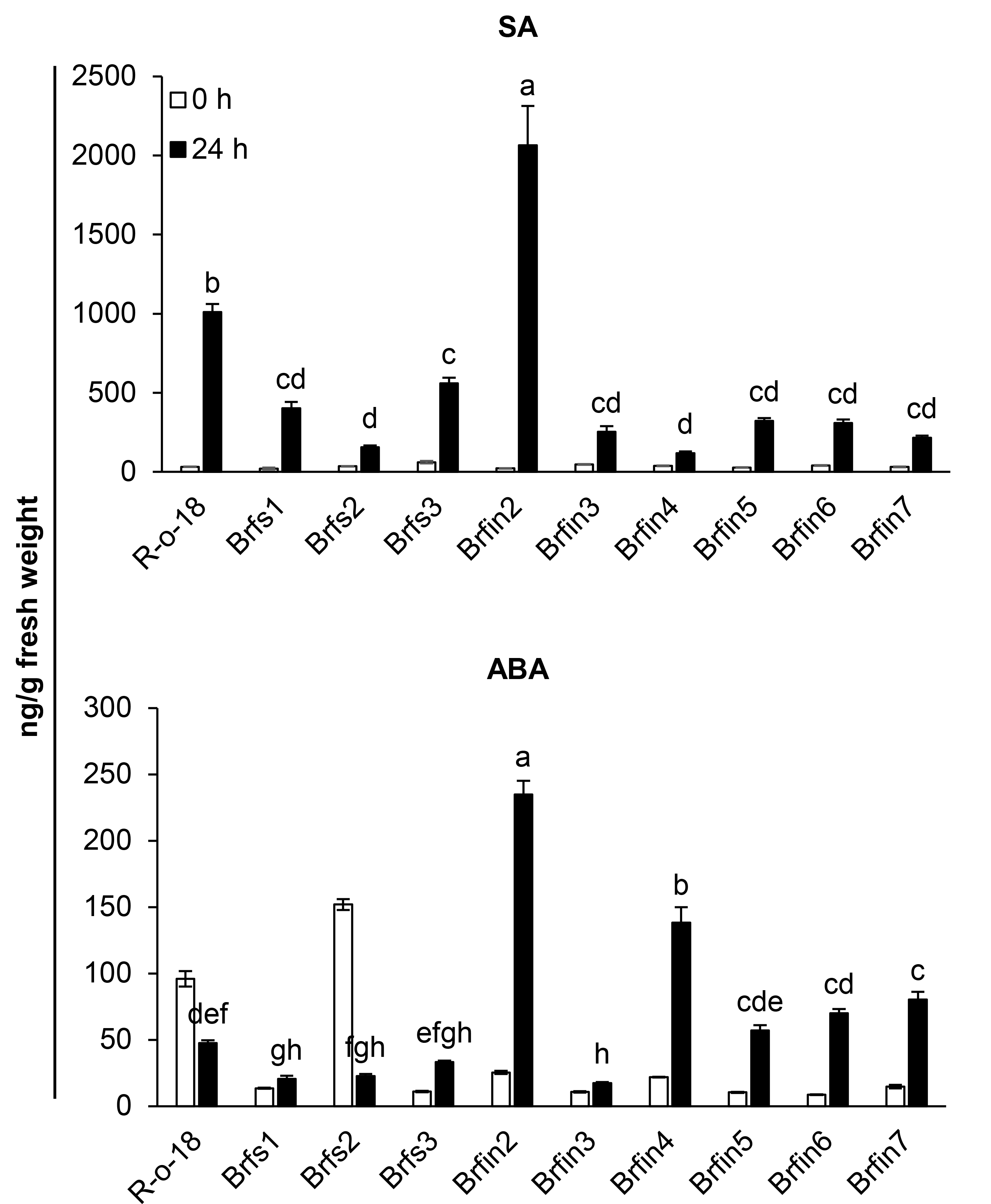

Supplement: Supplementary file 1 [file Image_1.jpeg]

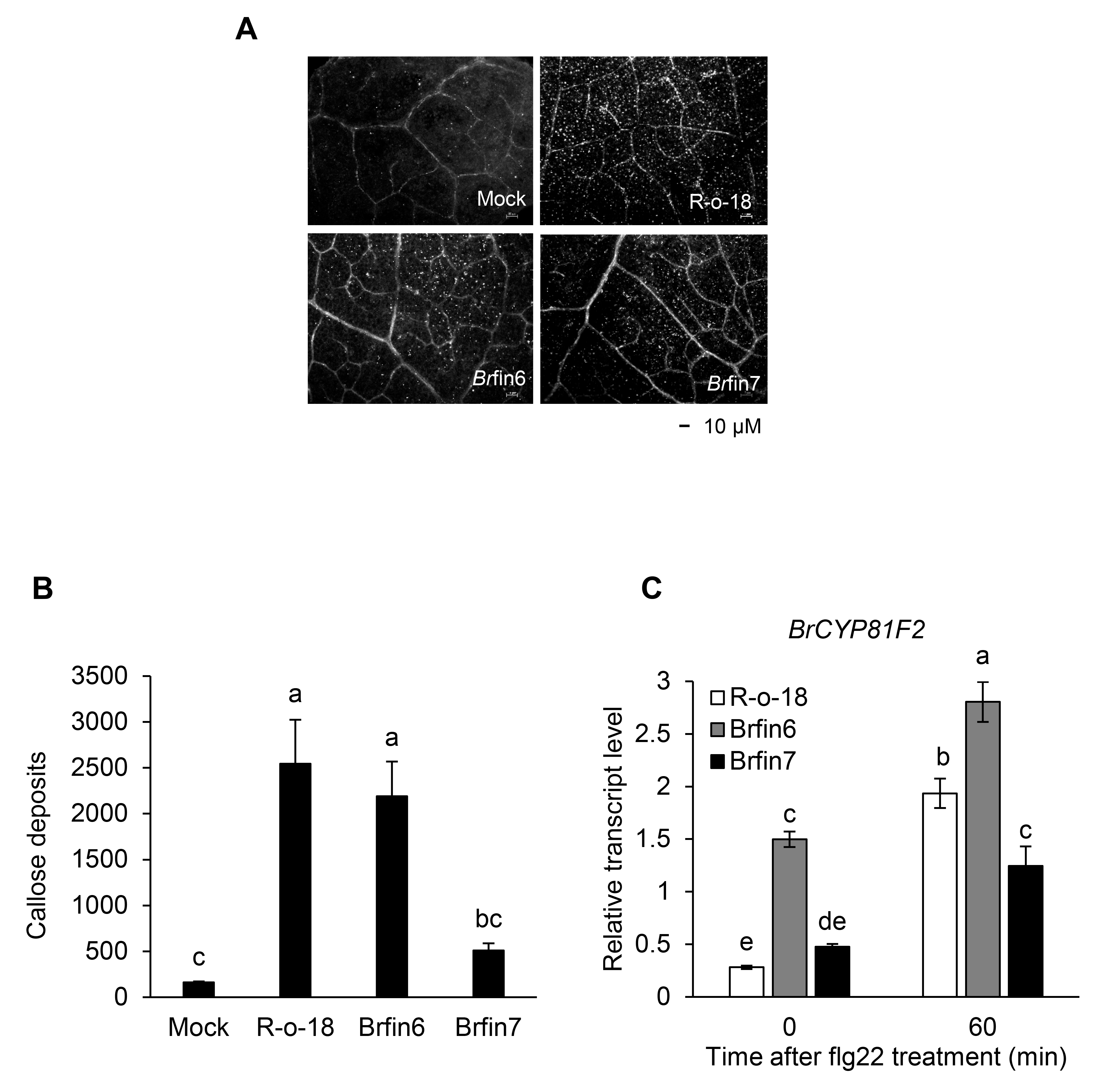

Supplement: Supplementary file 2 [file Image_2.jpeg]

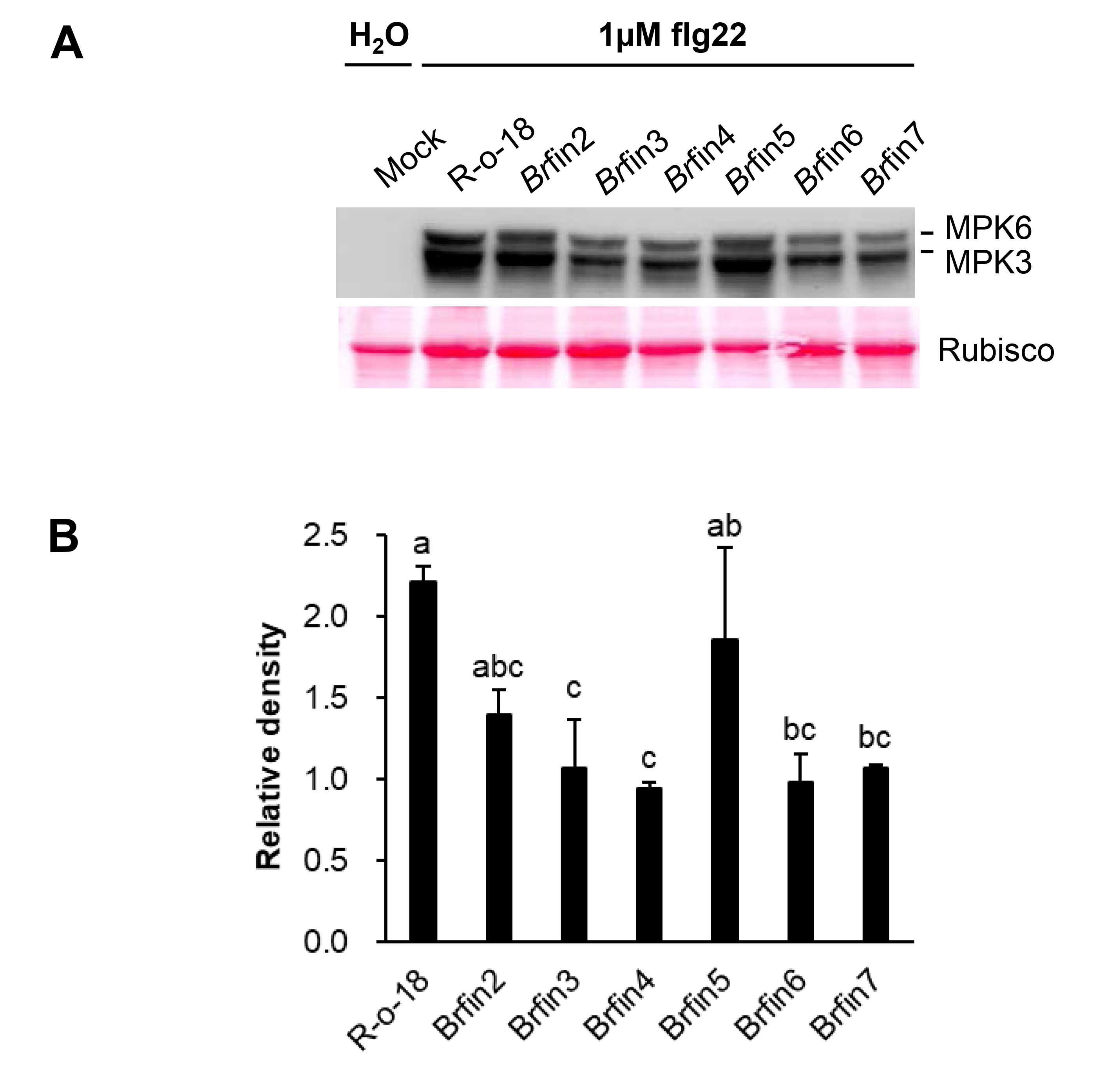

Supplement: Supplementary file 3 [file Image_3.jpeg]
