# Supplementary material for: Jasmonate regulates plant resistance to Pectobacterium brasiliense by inducing indole glucosinolate biosynthesis
Source: Front Plant Sci. 2022 Sep 29;13:964092. doi: 10.3389/fpls.2022.964092 (PMC9559233; doi:10.3389/fpls.2022.964092)
Supplement: Supplementary file 4 [file Table_1.docx]

Supplementary Material

**Supplementary Table 1.** Primers used for quantitative reverse-transcription PCR (qRT-PCR)

| **Primers** |  | **Primer sequences (5'-3')** | **Reference**  **Gene ID** |
| --- | --- | --- | --- |
| *BrACT2* | F | ACCCAAAGGCCAACAGAGAG | (Abe et al., 2011) |
|  | R | CTGGCGTAAAGGGAGAGAACA | Bra022356 |
| *BrWRKY33* | F | CAAACAGACTGGCACAAG | (Chen et al., 2016) |
|  | R | AGCGTAACCAATAGACCC | Bra005104 |
| *BrWRKY40* | F | TGTAGCTTGGCTAAGCCTGT |  |
|  | R | CCGGTAACAGCTGCTGCTA | Bra035148 |
| *BrNHL10* | F | TACGACAGGATCGACGCTCA |  |
|  | R | GTAACTCTCCGGCGTTGAACA | Bra017272 |
| *BrCYP81F2* | F | CGTCGTATCCACCAATTTTGAG |  |
|  | R | GTCCGAGATAGACGTTAAGATC | Bra006830 |
| *BrMYB51* | F | CAATTATAACGAGTGATGATGATCTTG |  |
|  | R | GTAGTAGTAGTAGTAAGTGGGCCACCA | Bra016553 |
| *BrST5a* | F | TCTGCAGCTACCCGAAAAC | (Seo et al., 2016) |
|  | R | GCGAAGTCTATCTCCACGTAAG | Bra008132 |
| *BrCYP81F4* | F | GCAGATACGTTCAAGAAACTCATT |  |
|  | R | ATTTCCTCTACACTCATCAAGCAG | Bra010598 |
| *BrIGMT1* | F | AAAGATGTGAAAGTGTTGGTTGAT |  |
|  | R | ATCATGAAGTATGCGTTTCAAAAT | Bra012270 |

**References**

Abe, H., Narusaka, Y., Sasaki, I., Hatakeyama, K., Shin-I, S., Narusaka, M., et al. (2011). Development of Full-Length cDNAs from Chinese Cabbage (Brassica rapa Subsp pekinensis) and Identification of Marker Genes for Defence Response. *DNA Research* 18(4)**,** 277-289. doi: 10.1093/dnares/dsr018.

Chen, J.J., Pang, W.X., Chen, B., Zhang, C.Y., and Piao, Z.Y. (2016). Transcriptome Analysis of Brassica rapa Near-lsogenic Lines Carrying Clubroot-Resistant and -Susceptible Alleles in Response to Plasmodiophora brassicae during Early Infection. *Frontiers in Plant Science* 6. doi: ARTN 1183 10.3389/fpls.2015.01183.

Seo, M.S., Jin, M., Chun, J.H., Kim, S.J., Park, B.S., Shon, S.H., et al. (2016). Functional analysis of three BrMYB28 transcription factors controlling the biosynthesis of glucosinolates in Brassica rapa. *Plant Molecular Biology* 90(4-5)**,** 503-516. doi: 10.1007/s11103-016-0437-z.
